# Postoperative Body Image Perceptions and Mental Health Outcomes in Adults Undergoing Bariatric Surgery: A Cross-Sectional Study

**DOI:** 10.7759/cureus.87607

**Published:** 2025-07-09

**Authors:** Mousa A Alamri, Yasir A Alharbi, Hatem H Alharbi, Ali A Jumah

**Affiliations:** 1 General Surgery, Prince Sultan Armed Forces Hospital, Madinah, SAU; 2 General Surgery, King Fahad Hospital, Madinah, SAU; 3 General Surgery, Royal Commission Medical Center, Madinah, SAU

**Keywords:** bariatric surgery, body image, depression, excess skin, mental health, obesity, postoperative care, psychosocial outcomes, quality of life, saudi arabia

## Abstract

Background: Bariatric surgery is the most effective treatment for severe obesity, offering durable weight loss and improvement in comorbidities. However, its psychosocial outcomes, particularly in non-Western settings, are less understood. This study assessed postoperative body image perceptions and mental health outcomes among adults in Saudi Arabia and identified factors associated with depressive symptoms and psychosocial functioning.

Methods: We conducted a cross-sectional survey between January and May 2025 at three tertiary hospitals in Saudi Arabia. Adults (≥18 years) who had undergone bariatric surgery ≥ six months prior were recruited (N = 240). Participants completed a validated, self-administered questionnaire covering body image, mental health, social activity, and overall satisfaction. Multivariable logistic regression identified independent predictors of moderate-to-severe depressive symptoms.

Results: Of the 240 participants, 148 (61.7%) were female and most (n = 144; 60.0%) were aged 26-45 years. Sleeve gastrectomy was the most common procedure (n = 178; 74.2%). A total of 150 participants (62.5%) reported satisfaction with their postoperative appearance, while 52 (21.7%) were dissatisfied or very dissatisfied. Excess skin was a concern for 88 participants (36.7%). Moderate-to-severe depressive symptoms were reported by 84 participants (35.0%), and 98 (40.8%) experienced anxiety on more than half the days in the prior two weeks. Social activity increased postoperatively in 114 participants (47.5%), while 66 (27.5%) reported reduced engagement. Interest in psychological support was expressed by 140 participants (58.4%). Body dissatisfaction was significantly associated with depressive symptoms: 46 of 88 dissatisfied participants (52.3%) met depression criteria versus 12 of 86 (14.0%) with low dissatisfaction (P < 0.001). Independent predictors of depression included body dissatisfaction (adjusted odds ratio (aOR), 3.46; 95% CI, 1.94-6.17), excess skin concerns (aOR, 2.21; 95% CI, 1.24-3.93), decreased social activity (aOR, 2.94; 95% CI, 1.61-5.35), and female gender (aOR, 1.82; 95% CI, 1.05-3.14).

Conclusions: Despite weight loss benefits, a substantial subset of bariatric patients in Saudi Arabia continue to face body dissatisfaction and psychological distress. Integrating culturally tailored mental health support into postoperative care is essential to optimize long-term psychosocial outcomes.

## Introduction

Bariatric surgery is widely recognized as the most effective long-term treatment for severe obesity, offering substantial and sustained weight loss as well as improvement in obesity-related comorbidities such as type 2 diabetes, hypertension, and sleep apnea [1-4]. While its physical and metabolic benefits are well documented, the psychological and psychosocial consequences of bariatric surgery have received comparatively less consistent attention in the literature [4-7].

Patients often pursue bariatric surgery not only for health reasons but also with the hope of achieving improved self-image, emotional well-being, and social functioning [1-3]. Although many report enhanced mood and self-esteem postoperatively, emerging evidence suggests that a significant subset of patients continues to experience body dissatisfaction, depressive symptoms, and anxiety even after substantial weight loss [5,6]. Among the most commonly reported issues is distress related to excess skin or unmet aesthetic expectations, which may undermine quality of life and psychological recovery.

Furthermore, mental health support is frequently underutilized or inconsistently integrated into postoperative care, especially in non-Western settings, where cultural and social norms around body image, stigma, and mental health may differ significantly [5-9]. Data on the long-term psychosocial outcomes of bariatric surgery in Middle Eastern populations remain limited, and there is a lack of structured, region-specific research examining the links between postoperative body image, mental health, and satisfaction.

This study aimed to evaluate body image perceptions and mental health status in adults who had undergone bariatric surgery in Saudi Arabia and to identify factors associated with depressive symptoms and psychosocial outcomes. Understanding these relationships may inform more comprehensive and culturally tailored postoperative care strategies.

## Materials and methods

Study design and setting

We conducted a cross-sectional, survey-based study to assess body image perceptions and mental health outcomes among adults who had undergone bariatric surgery in Saudi Arabia. The study was conducted between January and May 2025 across three tertiary care hospitals with established bariatric surgery units, including both governmental and private institutions. All data were collected anonymously through a structured, self-administered questionnaire.

Participants

Eligible participants were adults aged 18 years or older who had undergone bariatric surgery (including sleeve gastrectomy, Roux-en-Y gastric bypass, or adjustable gastric banding) at least six months before the time of recruitment. Patients were excluded if they had a diagnosis of active psychosis or cognitive impairment, or if they declined to participate. A total of 240 participants were included, selected through convenience sampling from outpatient follow-up clinics and patient support groups.

Survey instrument

Data were collected using a closed-ended, multiple-choice questionnaire developed specifically for this study (see Appendix). The survey was organized into four domains. The first domain captured sociodemographic and clinical characteristics, including age group, gender, marital status, education level, type of surgery, and time since surgery. The second domain focused on body image outcomes, with questions addressing satisfaction with appearance, mirror avoidance, confidence in wearing clothing, and concerns related to excess or loose skin. The third domain assessed mental health and emotional functioning through items related to depressive symptoms, anxiety levels, social activity, mood changes, and interest in psychological support. The fourth domain evaluated overall satisfaction and quality of life, including participants’ perceptions of life improvement following surgery and their likelihood of recommending bariatric surgery to others.

The questionnaire was originally constructed in English and then translated into Arabic by bilingual health professionals. It was subsequently back-translated by an independent translator to ensure content validity. Face validity and cultural appropriateness were assessed by a panel of a bariatric surgeon and two psychologists. A pilot test was conducted on 15 participants (not included in the final sample) to ensure clarity and internal consistency. Minor modifications were made based on feedback.

Data collection procedures

Eligible participants were approached during routine follow-up visits or via secure links shared through patient advocacy groups. After confirming eligibility and obtaining informed consent, participants completed the survey independently using paper or electronic forms, based on preference. The average completion time was approximately 8-10 minutes. To ensure confidentiality, no identifying information was collected. Participation was voluntary and did not affect clinical care.

Statistical analysis

Descriptive statistics were used to summarize the demographic and clinical characteristics of the sample. Categorical variables were presented as counts and percentages. Group comparisons were conducted using the chi-square test for categorical variables. For the analysis of factors associated with moderate-to-severe depressive symptoms, body dissatisfaction, and psychosocial outcomes, multivariate logistic regression models were employed. Variables included in the multivariable model were selected a priori based on clinical relevance and bivariate associations with a P-value <0.10.

Odds ratios (ORs) and 95% confidence intervals (CIs) were reported. Goodness-of-fit for the final model was assessed using the Hosmer-Lemeshow test. Missing data were minimal (<5%) and were handled using listwise deletion. All statistical tests were two-sided, and a P-value <0.05 was considered statistically significant. All analyses were performed using IBM SPSS Statistics for Windows, version 28.0 (IBM Corp., Armonk, NY, USA).

## Results

Participant characteristics

A total of 240 participants who had undergone bariatric surgery were included in the study. The majority were between 26 and 45 years of age, with 78 participants (32.5%) aged 26-35 years and 66 (27.5%) aged 36-45 years. A smaller proportion were aged 18-25 years (n = 28, 11.7%) or over 55 years (n = 24, 10.0%). Most participants were female (n = 148, 61.7%), and 146 (60.8%) were married. Regarding education, 112 (46.7%) held a bachelor’s degree, and 58 (24.2%) had completed a master’s degree or higher.

Sleeve gastrectomy was the most common procedure (n = 178, 74.2%), followed by Roux-en-Y gastric bypass (n = 42, 17.5%). Time since surgery varied: 78 participants (32.5%) had undergone surgery one to two years prior, 68 (28.3%) were two to five years postoperative, and 56 (23.3%) were within six months to one year of surgery (Table 1).

**Table 1:** Baseline characteristics of participants (N = 240). Shown are the sociodemographic and clinical characteristics of the study participants, including age group, gender, marital status, education level, time since bariatric surgery, and type of surgical procedure performed. Data are presented as number of participants and corresponding percentages.

Characteristic		n (%)
Age group (years)	18–25	28 (11.7%)
	26–35	78 (32.5%)
	36–45	66 (27.5%)
	46–55	44 (18.3%)
	>55	24 (10.0%)
Gender	Male	92 (38.3%)
	Female	148 (61.7%)
Marital status	Single	62 (25.8%)
	Married	146 (60.8%)
	Divorced	22 (9.2%)
	Widowed	10 (4.2%)
Education level	Primary	18 (7.5%)
	Secondary	52 (21.7%)
	Bachelor’s	112 (46.7%)
	Master’s or higher	58 (24.2%)
Time since surgery	6 months–1 year	56 (23.3%)
	1–2 years	78 (32.5%)
	2–5 years	68 (28.3%)
	>5 years	38 (15.8%)
Surgery type	Sleeve gastrectomy	178 (74.2%)
	Roux-en-Y gastric bypass	42 (17.5%)
	Adjustable gastric band	12 (5.0%)
	Other/not sure	8 (3.3%)

Body image outcomes

Participants expressed varied levels of satisfaction with their body image following surgery. A total of 150 participants (62.5%) reported being either very satisfied (n = 66, 27.5%) or satisfied (n = 84, 35.0%) with their postoperative body appearance, while 34 (14.2%) were dissatisfied and 18 (7.5%) were very dissatisfied. When asked about mirror avoidance, 70 participants (29.2%) admitted to sometimes avoiding mirrors, and 54 (22.5%) reported doing so often or always.

Confidence in wearing tight or revealing clothing increased in 134 participants (55.9%) following surgery, though 22 (9.2%) reported feeling much less confident than before. Concerns related to excess or loose skin were common: 88 participants (36.7%) reported being very or extremely bothered by this issue (Table 2).

**Table 2:** Body image perceptions and related outcomes after bariatric surgery (N = 240). This table presents participant responses related to postoperative body image, including satisfaction with appearance, behaviors related to mirror avoidance, confidence in wearing tight or revealing clothing, and the degree of distress caused by loose or excess skin. Data are shown as the number of participants and corresponding percentages for each response category.

Survey item		n (%)
Satisfied with body appearance	Very satisfied	66 (27.5%)
	Satisfied	84 (35.0%)
	Neutral	38 (15.8%)
	Dissatisfied	34 (14.2%)
	Very dissatisfied	18 (7.5%)
Avoid looking in the mirror	Never	52 (21.7%)
	Rarely	64 (26.7%)
	Sometimes	70 (29.2%)
	Often	34 (14.2%)
	Always	20 (8.3%)
Confidence in wearing tight clothes	Much more confident	58 (24.2%)
	Slightly more confident	76 (31.7%)
	No change	48 (20.0%)
	Slightly less confident	36 (15.0%)
	Much less confident	22 (9.2%)
Bothered by loose/excess skin	Not at all	38 (15.8%)
	Slightly	48 (20.0%)
	Moderately	66 (27.5%)
	Very	54 (22.5%)
	Extremely	34 (14.2%)

Mental health and psychosocial functioning

Symptoms of low mood and anxiety remained prevalent postoperatively. In the two weeks before the survey, 84 participants (35.0%) reported feeling down or hopeless on more than half the days or nearly every day, and 98 participants (40.8%) reported experiencing anxiety with similar frequency. When asked to evaluate changes in mood since surgery, 140 participants (58.4%) noted some improvement, 58 (24.2%) reported much improvement, and 82 (34.2%) reported slight improvement, while 48 (20.0%) reported worsened mood.

Regarding social behavior, 114 participants (47.5%) became more socially active after surgery, though 66 (27.5%) reported decreased social engagement or avoidance of social situations altogether. When asked about interest in psychological support, 140 participants (58.4%) responded "definitely yes" (n = 76, 31.7%) or "probably yes" (n = 64, 26.7%) (Table 3).

**Table 3:** Mental health and psychosocial outcomes (N = 240).

Survey item		n (%)
Felt down or hopeless (last 2 weeks)	Not at all	70 (29.2%)
	Several days	86 (35.8%)
	More than half the days	50 (20.8%)
	Nearly every day	34 (14.2%)
Felt anxious or nervous (last 2 weeks)	Not at all	64 (26.7%)
	Several days	78 (32.5%)
	More than half the days	56 (23.3%)
	Nearly every day	42 (17.5%)
Mood change post surgery	Much improved	58 (24.2%)
	Slightly improved	82 (34.2%)
	No change	52 (21.7%)
	Slightly worse	30 (12.5%)
	Much worse	18 (7.5%)
Social activity	Much more active	40 (16.7%)
	Slightly more active	74 (30.8%)
	No change	60 (25.0%)
	Less active	44 (18.3%)
	Avoids social settings	22 (9.2%)
Desire for mental health support post surgery	Definitely yes	76 (31.7%)
	Probably yes	64 (26.7%)
	Not sure	42 (17.5%)
	Probably not	32 (13.3%)
	Definitely not	26 (10.8%)

Association between body image and depression symptoms

Higher levels of body dissatisfaction were significantly associated with increased symptoms of depression. Among participants who reported being very or extremely dissatisfied with their appearance (n = 88), 46 (52.3%) met the criteria for moderate to severe depressive symptoms, compared with only 12 (14.0%) of those reporting low dissatisfaction (n = 86). This relationship was statistically significant (P < 0.001) (Table 4).

**Table 4:** Association between body dissatisfaction and depression symptoms (N = 240). This table presents the relationship between levels of self-reported body dissatisfaction and the presence of moderate-to-severe depressive symptoms among participants. Depression severity was categorized based on responses to mood-related survey items. A significant association was observed, with higher levels of body dissatisfaction correlating with a greater likelihood of moderate-to-severe depression. P-value derived from chi-square test (df = 2).

Body dissatisfaction level	Moderate-to-severe depression (n = 84)	No/mild depression (n = 156)	P-value
Not at all/slightly (n = 86)	12 (14.0%)	74 (86.0%)	<0.001
Moderately (n = 66)	26 (39.4%)	40 (60.6%)	
Very/extremely (n = 88)	46 (52.3%)	42 (47.7%)	

Predictors of moderate-to-severe depression

In multivariable logistic regression analysis, several factors were independently associated with moderate-to-severe depression symptoms. Dissatisfaction with body appearance was the strongest predictor, with an adjusted odds ratio (aOR) of 3.46 (95% CI, 1.94-6.17; P < 0.001). Excess skin concerns were also associated with increased odds of depression (aOR, 2.21; 95% CI, 1.24-3.93; P = 0.007). Participants who reported decreased social activity or social avoidance had nearly three times the odds of experiencing significant depressive symptoms (aOR, 2.94; 95% CI, 1.61-5.35; P < 0.001). Female gender was also a significant predictor (aOR, 1.82; 95% CI, 1.05-3.14; P = 0.032), while age and time since surgery were not (Table 5).

**Table 5:** Multivariate logistic regression: predictors of moderate-to-severe depression after bariatric surgery (N = 240). This table shows adjusted odds ratios (ORs), 95% confidence intervals (CIs), and P-values for variables independently associated with moderate-to-severe depressive symptoms. The model included gender, age, body dissatisfaction, excess skin concerns, time since surgery, and social withdrawal. Marital status, education level, and type of surgery were included as covariates in the model. Model fit was acceptable (Hosmer–Lemeshow goodness-of-fit test, P = 0.68).

Predictor variable	Adjusted OR (95% CI)	P-value
Female gender	1.82 (1.05–3.14)	0.032
Age > 45 years	0.66 (0.36–1.20)	0.173
Dissatisfaction with body appearance	3.46 (1.94–6.17)	<0.001
Excess skin (very/extremely)	2.21 (1.24–3.93)	0.007
Time since surgery (>2 years)	0.78 (0.42–1.46)	0.438
Social withdrawal (less active/avoids)	2.94 (1.61–5.35)	<0.001

## Discussion

In this cross-sectional study of 240 adults who had undergone bariatric surgery in Saudi Arabia, we found that while the majority of patients reported improved body image and mood postoperatively, a substantial proportion continued to experience psychological distress, including body dissatisfaction, anxiety, and depressive symptoms. Notably, body dissatisfaction and concerns about excess skin were strongly associated with moderate-to-severe depressive symptoms, independent of age, gender, and time since surgery. These findings highlight the complex interplay between physical transformation and psychological adaptation following bariatric procedures.

Most participants reported improvements in body image satisfaction, clothing confidence, and social activity, consistent with prior research showing positive effects of bariatric surgery on self-perception and quality of life. However, nearly one in four participants reported ongoing dissatisfaction with their appearance, and more than one-third (n = 88, 36.7%) expressed being very or extremely bothered by excess or loose skin. These findings underscore that while weight loss may improve general body satisfaction, it does not uniformly eliminate appearance-related concerns. The persistence of body image distress may reflect a mismatch between patients’ preoperative expectations and postoperative reality, particularly in those who develop significant skin redundancy [8-11].

Despite physical improvements, 84 participants (35.0%) met the criteria for moderate-to-severe depressive symptoms, and nearly half (n = 98, 40.8%) reported frequent anxiety. While 58.4% (n = 140) described an improved mood since surgery, these self-reported benefits appear to coexist with considerable psychological burden. Importantly, over half the cohort expressed a desire for mental health support, suggesting unmet needs in postoperative care. These findings align with existing literature indicating that while bariatric surgery is associated with short-term improvements in mood, a subset of patients may develop or continue to experience psychiatric symptoms, especially beyond the first postoperative year [12-15].

The strong association between body dissatisfaction and depressive symptoms in our sample (OR, 3.46; 95% CI, 1.94-6.17) suggests that perceived body image, rather than absolute weight loss alone, may be a critical determinant of mental health outcomes after bariatric surgery. Concerns about loose skin also independently predicted depression (OR, 2.21), further emphasizing the need to address body contouring and aesthetic support as part of comprehensive postoperative care.

Our findings support a more holistic approach to bariatric aftercare. While surgery effectively addresses metabolic and weight-related outcomes, psychological follow-up is often fragmented or overlooked. Integrating mental health screening, patient education about realistic body image outcomes, and timely referral to counseling or reconstructive surgery evaluation may help mitigate the psychological burden identified in this study [14,16]. Given that social withdrawal was also associated with depression (aOR, 2.94), behavioral activation and structured peer support interventions may be particularly beneficial.

Strengths and limitations

The strengths of this study include its focus on both body image and mental health outcomes using a structured instrument grounded in validated tools, its relatively large sample size, and its relevance to real-world clinical populations in the Middle East, a region where such data remain limited. However, several limitations should be acknowledged.

First, the cross-sectional design precludes causal inference, and the observed associations may reflect bidirectional or confounded relationships. Second, the use of self-reported, non-diagnostic measures to assess depressive and anxiety symptoms limits the ability to generalize to clinically diagnosed disorders. Third, convenience sampling from tertiary centers may introduce selection bias, and the findings may not generalize to rural or underserved populations. Finally, the survey’s reliance on closed-ended, multiple-choice questions limited the depth of qualitative insight into patients' emotional experiences.

## Conclusions

In conclusion, while bariatric surgery is associated with improvements in body image and mood for many patients, a substantial proportion continues to experience psychological distress, particularly related to excess skin, persistent body dissatisfaction, and reduced social engagement. These factors are strongly associated with depressive symptoms and signal the need for a more comprehensive, multidisciplinary postoperative care model that integrates psychological assessment, aesthetic counseling, and targeted mental health support. Addressing the emotional and psychosocial dimensions of life after bariatric surgery is essential not only to enhance long-term patient satisfaction but also to ensure that the full therapeutic potential of surgical weight loss is realized.

## Appendices

**Table 6:** The survey questionnaire used in the study.

Section	Question	Response options
1. Sociodemographic and clinical information	1. Age group	☐ 18–25 ☐ 26–35 ☐ 36–45 ☐ 46–55 ☐ Over 55
	2. Gender	☐ Male ☐ Female
	3. Marital status	☐ Single ☐ Married ☐ Divorced ☐ Widowed
	4. Education level	☐ Primary ☐ Secondary ☐ Bachelor’s ☐ Master’s or higher
	5. Type of bariatric surgery	☐ Sleeve gastrectomy ☐ Roux-en-Y gastric bypass ☐ Adjustable gastric band ☐ Other/Not sure
	6. Time since surgery	☐ 6 months–1 year ☐ 1–2 years ☐ 2–5 years ☐ More than 5 years
2. Body image and appearance satisfaction	7. Satisfaction with current body appearance	☐ Very satisfied ☐ Satisfied ☐ Neutral ☐ Dissatisfied ☐ Very dissatisfied
	8. Do you avoid looking at yourself in the mirror?	☐ Never ☐ Rarely ☐ Sometimes ☐ Often ☐ Always
	9. Confidence in wearing tight or revealing clothing	☐ Much more confident ☐ Slightly more confident ☐ No change ☐ Slightly less confident ☐ Much less confident
	10. Bothered by loose or excess skin	☐ Not at all ☐ Slightly ☐ Moderately ☐ Very ☐ Extremely
3. Mental health and psychosocial functioning	11. Felt down, depressed, or hopeless (last 2 weeks)	☐ Not at all ☐ Several days ☐ More than half the days ☐ Nearly every day
	12. Felt anxious or nervous (last 2 weeks)	☐ Not at all ☐ Several days ☐ More than half the days ☐ Nearly every day
	13. Mood change compared to before surgery	☐ Much improved ☐ Slightly improved ☐ No change ☐ Slightly worse ☐ Much worse
	14. Change in social activity since surgery	☐ Much more active ☐ Slightly more active ☐ No change ☐ Less active ☐ I avoid social settings
	15. Interest in psychological support	☐ Definitely yes ☐ Probably yes ☐ Not sure ☐ Probably not ☐ Definitely not
4. Overall satisfaction and quality of life	16. Has your quality of life improved since surgery?	☐ Strongly agree ☐ Agree ☐ Neutral ☐ Disagree ☐ Strongly disagree
	17. Would you recommend bariatric surgery to others?	☐ Definitely yes ☐ Probably yes ☐ Not sure ☐ Probably not ☐ Definitely not

## References

[1] Kubik JF, Gill RS, Laffin M, Karmali S (2013). The impact of bariatric surgery on psychological health. J Obes.

[2] Nickel F, Schmidt L, Bruckner T, Büchler MW, Müller-Stich BP, Fischer L (2017). Influence of bariatric surgery on quality of life, body image, and general self-efficacy within 6 and 24 months—a prospective cohort study. Surg Obes Relat Dis.

[3] Legenbauer T, Müller A, de Zwaan M, Herpertz S (2019). Body image and body avoidance nine years after bariatric surgery and conventional weight loss treatment. Front Psychiatry.

[4] Mauro MF, Papelbaum M, Brasil MA, Carneiro JR, Luiz RR, Hiluy JC, Appolinario JC (2024). Mental health and weight regain after bariatric surgery: associations between weight regain and psychiatric and eating-related comorbidities. Arch Endocrinol Metab.

[5] Buer L, Kvalem IL, Bårdstu S, Mala T (2022). Comparing bariatric surgery patients who desire, have undergone, or have no desire for body contouring surgery: a 5-year prospective study of body image and mental health. Obes Surg.

[6] Song A, Fernstrom MH (2008). Nutritional and psychological considerations after bariatric surgery. Aesthet Surg J.

[7] Jumbe S, Hamlet C, Meyrick J (2017). Psychological aspects of bariatric surgery as a treatment for obesity. Curr Obes Rep.

[8] Wimmelmann CL, Dela F, Mortensen EL (2014). Psychological predictors of mental health and health-related quality of life after bariatric surgery: a review of the recent research. Obes Res Clin Pract.

[9] Singh D, Zahiri HR, Janes LE, Sabino J, Matthews JA, Bell RL, Thomson JG (2012). Mental and physical impact of body contouring procedures on post-bariatric surgery patients. Eplasty.

[10] Butt M, Su L, Rigby A (2022). Associations of use of social media and psychopathology and body image in pre- and post-surgical bariatric samples: a cross-sectional analysis. Obes Surg.

[11] Wallace L, Horecki EK, Helm MC, Higgins RM, Gould JC, Lak K, Kindel TL (2019). Buyer's remorse: what predicts post-decision dissonance after bariatric surgery?. Surg Obes Relat Dis.

[12] Dunford A, Ivezaj V, Grilo CM (2024). Shape discrepancy, weight bias internalization, and eating-disorder psychopathology in patients with loss-of-control eating after bariatric surgery. Surg Obes Relat Dis.

[13] Geller S, Levy S, Goldzweig G (2019). Psychological distress among bariatric surgery candidates: the roles of body image and emotional eating. Clin Obes.

[14] Müller A, Mitchell JE, Sondag C, de Zwaan M (2013). Psychiatric aspects of bariatric surgery. Curr Psychiatry Rep.

[15] Greenberg I, Perna F, Kaplan M, Sullivan MA (2005). Behavioral and psychological factors in the assessment and treatment of obesity surgery patients. Obes Res.

[16] Lundin Kvalem I, Gabrielsen L, Eribe I, Kristinsson JA, Mala T (2022). Predicting satisfaction with outcome and follow-up care 5 years after bariatric surgery: a prospective evaluation. Obes Sci Pract.

